# Revealing Chronic Granulomatous Disease in a Patient With Williams-Beuren Syndrome Using Whole Exome Sequencing

**DOI:** 10.3389/fimmu.2021.778133

**Published:** 2021-11-04

**Authors:** Adiratna Mat Ripen, Mei Yee Chiow, Prakash Rao Rama Rao, Saharuddin Bin Mohamad

**Affiliations:** ^1^ Primary Immunodeficiency Unit, Allergy and Immunology Research Centre, Institute for Medical Research, National Institutes of Health, Ministry of Health Malaysia, Selangor, Malaysia; ^2^ Institute of Biological Sciences, Faculty of Science, University of Malaya, Kuala Lumpur, Malaysia; ^3^ Pediatrics Department, Keningau Hospital, Ministry of Health Malaysia, Sabah, Malaysia; ^4^ Centre of Research in Systems Biology, Structural Bioinformatics and Human Digital Imaging (CRYSTAL), University of Malaya, Kuala Lumpur, Malaysia

**Keywords:** whole exome sequencing (WES), chronic granulomatous disease (CGD), Williams-Beuren syndrome (WBS), copy number variation (CNV), blended phenotypes, dual molecular diagnosis

## Abstract

Blended phenotypes exhibited by a patient may present a challenge to the establishment of diagnosis. In this study, we report a seven-year-old Murut girl with unusual features of Williams-Beuren syndrome (WBS), including recurrent infections and skin abscesses. Considering the possibility of a second genetic disorder, a mutation screening for genes associated with inborn errors of immunity (IEI) was conducted using whole exome sequencing (WES). Analysis of copy number variations (CNVs) from the exome data revealed a 1.53Mb heterozygous deletion on chromosome 7q11.23, corresponding to the known WBS. We also identified a biallelic loss of *NCF1*, which indicated autosomal recessive chronic granulomatous disease (CGD). Dihydrorhodamine (DHR) flow cytometric assay demonstrated abnormally low neutrophil oxidative burst activity. Coamplification of *NCF1* and its pseudogenes identified a GT-deletion (ΔGT) at the start of exon 2 in *NCF1* (NM_000265.7: c.75_76delGT: p.Tyr26Hisfs*26). Estimation of *NCF1*-to-*NCF1* pseudogenes ratio using ΔGT and 20-bp gene scans affirmed nil copies of *NCF1* in the patient. While the father had a normal ratio of 2:4, the mother had a ratio of 1:5, implicating the carrier of ΔGT-containing *NCF1*. Discovery of a 7q11.23 deletion involving one *NCF1* allele and a ΔGT in the second *NCF1* allele explained the coexistence of WBS and CGD in our patient. This study highlights the capability of WES to establish a molecular diagnosis for a case with blended phenotypes, enabling the provision of appropriate prophylactic treatment.

## Introduction

Williams-Beuren syndrome (WBS) (OMIM ID: 194050) is a contiguous gene deletion syndrome inherited in autosomal dominant pattern. Most cases occurred sporadically with an estimated prevalence of 1/7,500 to 1/20,000 live births (1, 2). Patients with WBS typically exhibited distinct facial appearance, cardiovascular abnormalities and developmental delay (3). Other features including idiopathic infantile hypercalcemia, gastrointestinal problems, musculoskeletal defects, hypothyroidism and hernias were noted in some patients (4). This genetic disorder is caused by a heterozygous deletion of 1.5-1.8Mb on chromosome 7q11.23 that encodes approximately 26 to 28 genes (5). Phenotypic variability between WBS patients may complicate the establishment of diagnosis based on clinical symptoms. Hence, a comprehensive genetic testing is necessary to confirm the diagnosis, particularly in cases with unusual manifestations.

Our study involved a seven-year-old patient with blended phenotypes. The patient manifested typical WBS features such as aortic stenosis, inguinal hernia, subclinical hypothyroidism and learning difficulty with developmental delay. Nevertheless, she also had recurrent infections, diarrhea and skin abscesses since the age of one year. As WBS patients are not commonly associated with immune disorders, we suspected the possibility of a primary immunodeficiency. Primary immunodeficiencies, recently termed as inborn errors of immunity (IEI) are characterized by increased susceptibility to infections, autoimmune diseases, autoinflammatory disorders, allergies and malignancies (6). These disorders are caused by monogenic mutations in at least 430 genes (6, 7). Diagnosing IEI based on the clinical and immunological abnormalities can be challenging owing to their phenotypic and genetic heterogeneity (8). Since the application of next-generation sequencing (NGS), the molecular diagnostics of IEI was revolutionized, facilitating the continuous discovery of IEI-associated genes (6, 9).

Previously, we utilized whole exome sequencing (WES) to diagnose patients suspected with IEI and obtained molecular diagnoses in 46.7% of the cohort (10). Among the three NGS approaches, whole genome sequencing (WGS) has the highest diagnostic capacity compared to targeted gene panel and WES. Still, WGS is minimally used in the clinical settings because of its high cost and large data output. Although a targeted gene panel generates more manageable data, it restricts the identification of novel disease-causing genes. In light of the affordable cost and computational requirements, WES is reasonable for both diagnostic and research purposes of IEI (11). The likelihood of encountering incidental findings and variants of uncertain significance increases as the NGS coverage expands. Thus, NGS data should be interpreted meticulously to ensure accurate reporting of deleterious mutations. Herein, we identified the genetic etiology responsible for the unexpected features of WBS in our patient using WES.

## Materials and Methods

### Study Subject

A seven-year-old Murut girl with WBS caused by a heterozygous deletion (cytogenetic analysis not shown) was recruited by the Institute for Medical Research. The patient was presented with unexpected phenotypes of WBS, i.e., multiple severe infections and abscess formation, raising clinical suspicion of a cooccurring genetic disorder. Written informed consent was collected from the patient and her parents upon blood sample collection. The study received approval from the Medical Research and Ethics Committee, Ministry of Health Malaysia (KKM/NIHSEC/P16-837) and adhered to the Declaration of Helsinki.

### Exome Sequencing and Bioinformatics Analysis

Genomic DNA was isolated from peripheral blood mononuclear cells using QIAamp DNA Blood Mini Kit (Qiagen, Germany) according to the manufacturer’s protocol. Exome capture was performed using Agilent SureSelect Human All Exon V5 (Agilent, USA) with target size of 50Mb. Paired-end reads of 101 base pairs (bp) were generated from a HiSeq 4000 sequencer (Illumina, USA) at mean coverage of 100x. Burrows-Wheeler Aligner-maximal exact matches (BWA-MEM) was used to align the sequencing reads to the human reference genome GRCh38 (12). Mapped reads were subjected to soft clipping of adapter sequences, duplicate marking and read sorting using Picard tools. Genome Analysis Toolkit (GATK) was used for base quality score recalibration followed by variant calling. Variants including single nucleotide variants (SNVs) and short insertions or deletions (indels) were annotated using web-based ANNOVAR.

### Variant Prioritization

Annotated variants were primarily filtered against the known IEI-associated genes documented by the International Union of Immunological Societies in 2019 and 2021 (6, 7). Exonic and splice site variants resulting in missense, nonsense, frameshift and nonframeshift mutations were retained. Rare variants with allele frequency of 0.0001 or less as reported by Genome Aggregation Database (gnomAD) were further analyzed. The functional impact of the variants was evaluated using *in silico* pathogenicity prediction tools, namely Sorting Intolerant From Tolerant (SIFT), Polymorphism Phenotyping v2 (PolyPhen-2) and MutationTaster (13–15). Variants with tolerated or benign impact predicted by more than one variant effect predictor were excluded from analysis. Mode of inheritance and genotype-phenotype correlation were determined through extensive literature search. The variants were interpreted according to the standards and guidelines of American College of Medical Genetics and Genomics (ACMG) (16).

### Copy Number Variation (CNV) Analysis

ExomeDepth was used to detect CNVs from WES data *via* read depth approach (17). Read counts were generated from the BAM file containing aligned reads. An optimized reference set was built by selecting the most correlated samples with the study subject from the in-house exomes. Each exon was assigned with a likelihood value that designates one of the copy number variable states, namely deletion, normal and duplication. The likelihood values across multiple exons were merged using hidden Markov model. Bayes factor, which represents the likelihood ratio of CNV to normal copy number was calculated. In this regard, CNV with a high Bayes factor is more likely to be true positive. CNVs shared by the patient and control were excluded from analysis. CNVs on autosomes were interpreted based on findings reported in Database of Chromosomal Imbalance and Phenotype in Humans Using Ensembl Resources (DECIPHER) and Database of Genomic Variants (DGV) (18, 19). The potential disease-causing CNV was plotted to show the ratio between the observed and expected read depth.

### Dihydrorhodamine (DHR) Flow Cytometric Assay

Neutrophil oxidative burst activity was evaluated using Phagoburst™ kit (Orpegen Pharma, Germany) according to the manufacturer’s instructions. The heparinized whole blood sample was incubated with a stimulant, protein kinase C activator phorbol 12-myristate 13-acetate (PMA) at 37°C. A negative control was prepared by substituting the stimulant with wash solution. After 10 minutes of incubation, nonfluorescent dihydrorhodamine 123 substrate solution was added to every sample. Oxidation of dihydrorhodamine 123 to fluorescent rhodamine 123 upon reaction with reactive oxygen species (ROS) was measured by a BD FACSCanto™ II flow cytometer (Becton Dickinson, USA) using FlowJo™ v10. Neutrophils were gated to analyze the percentage of cells producing ROS during unstimulated and PMA-stimulated conditions.

### Genomic DNA Amplification and Sequencing

Polymerase chain reaction using published primers 2LB2 and 2RB2 was performed to screen for the GT-deletion (ΔGT) at the beginning of exon 2 in *NCF1* (20). The amplicons were sequenced bidirectionally using BigDye™ Terminator v3.1 Cycle Sequencing Kit (Applied Biosystems, USA) on a 3730xl DNA Analyzer (Applied Biosystems, USA). FinchTV was used to visualize the sequence chromatograms.

### ΔGT and 20-bp Gene Scans

Gene scans were performed to estimate the ratio of *NCF1* and its two pseudogenes (21). ΔGT gene scan targets the ΔGT in exon 2, while 20-bp gene scan targets both ΔGT in exon 2 and 20-bp copy in intron 2. The primers used for ΔGT gene scan were 6-FAM-labeled p47-ΔGT-fwd and p47-ΔGT-rev primers. Whereas for 20-bp gene scan, primers p47-ΔGT-fwd and HEX-labeled p47-20bp-rev were used. Separation of fluorescently labeled fragments was performed on a 3730xl DNA Analyzer (Applied Biosystems, USA). The ratio of *NCF1* and its pseudogenes was determined by dividing the peak heights displayed on PeakScanner.

## Results

### Case Presentation

The patient was the fifth child born to nonconsanguineous parents. Both parents and her four elder siblings were well with no known medical illnesses. Cytogenetic analysis was prompted before enrolment considering the characteristic facial features, revealing the diagnosis of WBS. However, this diagnosis did not explain the recurrence of infections and abscesses experienced by the patient since early childhood. Hence, she was recruited for further genetic analysis. She initially presented with fever, abdominal distension, diarrhea and limb stiffening at the age of one year. Blood culture and sensitivity test detected *Escherichia coli*, while cerebrospinal fluid fungal culture grew non-encapsulated yeast. She was treated as *Escherichia coli* sepsis and meningitis with ampicillin, metronidazole, cefepime, vancomycin, meropenem and amphotericin B. She was also noted to have abscesses on her left distal forearm and right popliteal area which were treated with cloxacillin. At the age of three years, she had prolonged fever with diarrhea and vomiting caused by *Salmonella typhi*, requiring a two-week course of cefuroxime. Until the age of five years, she was admitted for infectious diarrhea and treated with meropenem. One month later, she presented with a left knee abscess and right forearm cellulitis. Blood culture grew *Streptococcus* sp. In between the admissions, she also had multiple episodes of bronchopneumonia, requiring oxygen support and intravenous antibiotics. Laboratory screening tests encompassing full blood count assessment, lymphocyte subset enumeration and quantitation of immunoglobulin and complement levels were performed (**Table 1**). The test results showed no abnormalities except elevated IgE.

**Table 1:** Blood test results of the patient.

Parameter	(Unit)	Value	Age-matched reference range
Full blood count			
Red blood cells	(x10^12^/L)	5.0	4.0-6.0
Hemoglobin	(g/dL)	12.6	11.0-18.0
Hematocrit	(%)	43.1	35.0-60.0
MCV	(fL)	87.1	80.0-99.9
MCH	(pg)	25.5	27.0-31.0
MCHC	(g/dL)	29.3	33.0-37.0
RDW	(%)	16.5	11.6-13.7
White blood cells	(x10^9^/L)	14.3	4.5-10.5
Lymphocytes	(x10^9^/L)	6.7	1.2-3.4
Monocytes	(x10^9^/L)	2.6	0.1-0.6
Granulocytes	(x10^9^/L)	5.0	1.4-6.5
Platelets	(x10^9^/L)	784.0	150.0-450.0
Lymphocyte subsets			
CD3^+^ T cells	(x10^6^/L)	3,300	1,400-2,000
CD3^+^/CD4^+^ Th cells	(x10^6^/L)	1,328	700-1,000
CD3^+^/CD8^+^ Tc cells	(x10^6^/L)	2,211	600-900
CD19^+^ B cells	(x10^6^/L)	1,004	300-500
CD3^-^/CD16^+^56^+^ NK cells	(x10^6^/L)	222	200-600
Immunoglobulins			
IgG	(g/L)	18.70	5.20-15.60
IgA	(g/L)	2.27	0.54-3.60
IgM	(g/L)	3.51	0.13-2.40
IgE	(kU/L)	139	<40
Complements			
C3	(g/L)	2.00	0.50-0.90
C4	(g/L)	0.47	0.10-0.40

MCV, mean corpuscular volume; MCH, mean corpuscular hemoglobin; MCHC, mean corpuscular hemoglobin concentration; RDW, red cell distribution width; Th cells, helper T cells; Tc cells, cytotoxic T cells; NK cells, natural killer cells; Ig, immunoglobulin.

### Bioinformatics Interpretation of Exome Sequencing

**Figure 1:**
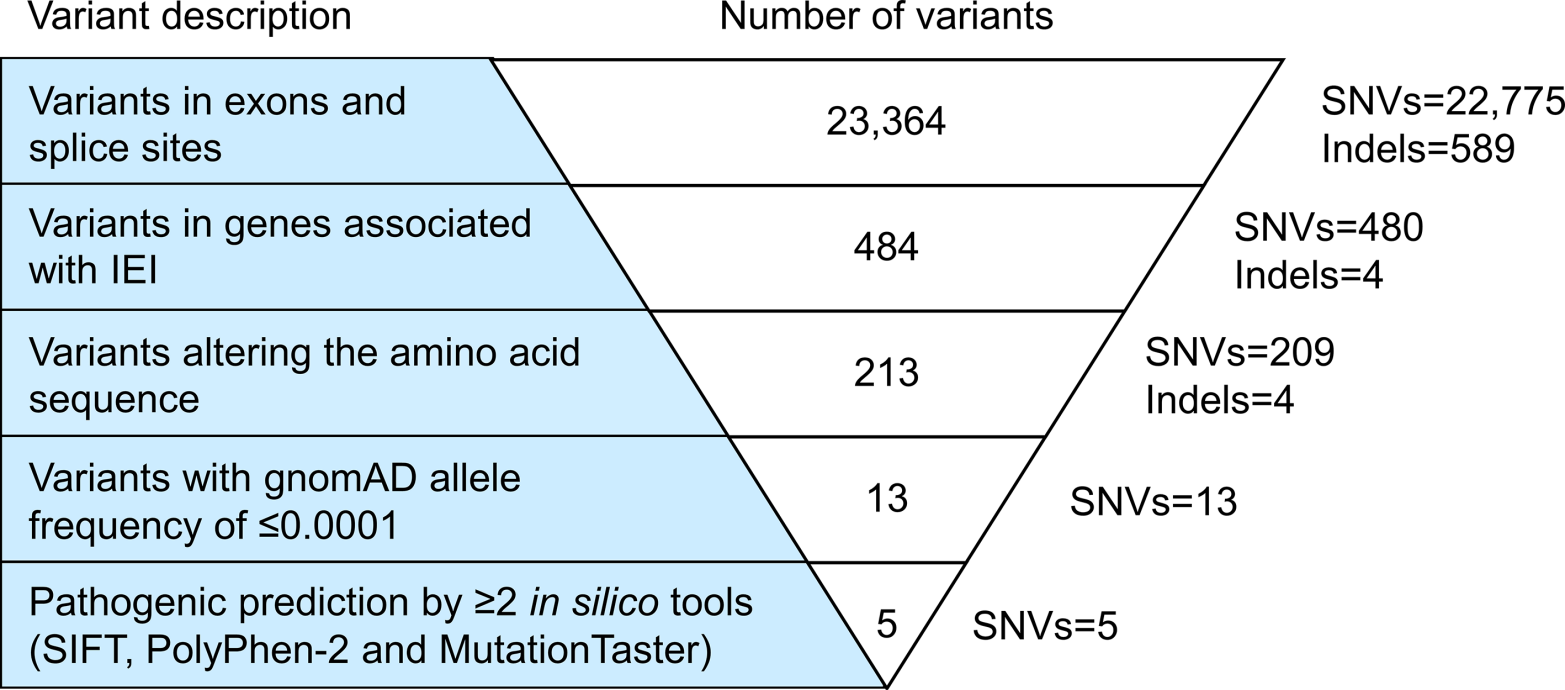
Variant filtering strategy. All variants harbored in the exonic regions and splice sites were filtered based on the known IEI-associated genes, variant class, allele frequency and variant functional impact. Five potential causative single nucleotide variants (SNVs) fulfilled the filtering criteria. However, the inheritance pattern and associated features of these genetic disorders did not match the zygosity and clinical symptoms of the patient respectively. IEI, inborn errors of immunity; SNVs, single nucleotide variants; Indels, small insertions or deletions.

Exome sequencing generated 67,606,668 paired-end reads with 99.5% of them properly paired and mapped to the human reference genome GRCh38. A total of 23,364 variants were detected in the exons (n=23,249) and splice sites (n=115) (**Figure 1**). These variants comprised 97.5% of SNVs and 2.5% of indels. After filtering against stringent criteria, the total variants were reduced to five potential disease-causing point mutations (**Table 2**). One SNV was identified in each of the five genes, namely *CFHR4*, *IL7R*, *CFTR*, *ERCC4* and *G6PD*. However, the inheritance pattern and associated features of these monogenic disorders were inconsistent with the patient’s zygosity and clinical presentation respectively.

**Table 2:** List of nonsynonymous single nucleotide variants (SNVs) found in the inborn errors of immunity (IEI) genes after variant filtering.

Chr	Gene	RefSeq:exon:nucleotide change:amino acid change	Inheritance pattern (zygosity)	dbSNP	*In silico* prediction				Allele frequency				ACMG classification
					SIFT	PolyPhen-2	MutationTaster	CADD phred	1000 Genomes All	1000 Genomes East Asian	gnomAD All	gnomAD East Asian	
1	*CFHR4*	NM_006684.5: exon5: c.620C>T: p.S207F	AR/AD (Het)	rs1296597051	D	PD	N	18.5	0	0	0	0	Likely benign
5	*IL7R*	NM_002185.5: exon4: c.460C>T: p.H154Y	AR (Hom)	rs199727195	D	PD	D	22.5	7.0E-05	3.0E-03	8.5E-05	1.0E-03	Uncertain significance
7	*CFTR*	NM_000492.4: exon14: c.1865G>A: p.G622D	AR (Het)	rs121908759	D	D	D	27.0	3.8E-04	1.0E-03	1.2E-04	5.6E-04	Pathogenic
16	*ERCC4*	NM_005236.3: exon11: c.2545C>G: p.Q849E	AR (Het)	rs374186605	T	PD	D	21.9	7.7e-05	2.0E-03	8.8E-05	1.1E-03	Likely benign
X	*G6PD*	NM_000402.4: exon6: c.682C>T: p.R228C	XL (Het)	rs137852330	D	D	D	27.6	2.7E-04	0	3.3E-05	7.2E-05	Likely benign

Chr, chromosome; AR, autosomal recessive inheritance; AD, autosomal dominant inheritance; X-linked inheritance; Het, heterozygous; Hom, homozygous; D, deleterious; T, tolerated; PD, possibly damaging; N, polymorphism; SIFT, Sorting Intolerant From Tolerant; PolyPhen-2, Polymorphism Phenotyping v2; CADD, Combined Annotation Dependent Depletion; gnomAD, genome aggregation database; ACMG, American College of Medical Genetics and Genomics.

### Biallelic Loss of *NCF1*

**Figure 2:**
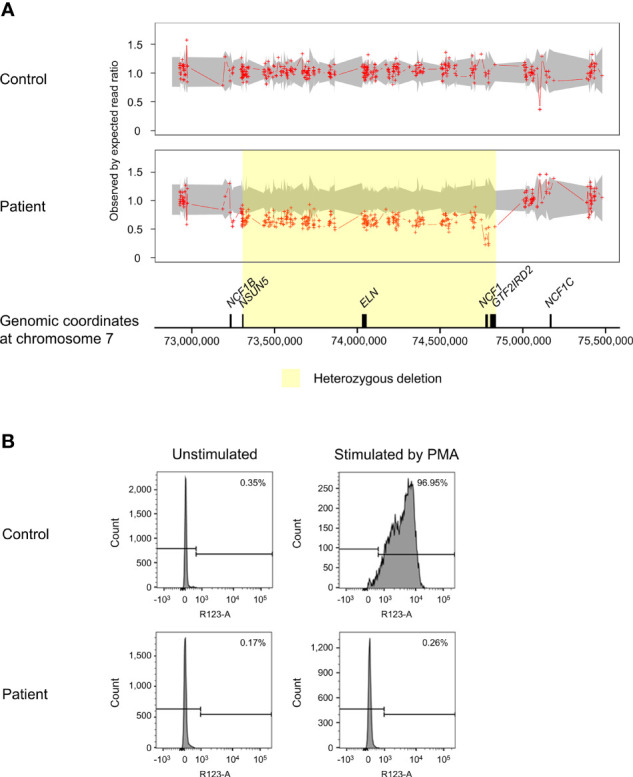
Copy number variation (CNV) analysis on chromosome 7q11.23 and assessment of NADPH oxidase activity. **(A)** The patient was found to have a biallelic deletion of *NCF1* localized within the 1.53Mb heterozygous deletion associated with Williams-Beuren syndrome (WBS) (shaded in yellow). The breakpoints of WBS deletion occurred from chromosome 7:73,303,201-74,836,526, affecting 27 genes. Biallelic loss of *NCF1* leads to autosomal recessive chronic granulomatous disease (CGD). Aortic stenosis observed in the patient is consistent with the haploinsufficiency of *ELN*. Ratio of 1.5 indicates one-copy duplication, 1.0 indicates normal copy state, 0.5 indicates one-copy deletion and 0 indicates two-copy deletion. Grey shade represents the 95% confidence interval of expected read ratio. Each red plus symbol indicates the genomic position being probed. Size of genes (black-filled boxes) was approximate and not drawn to scale. **(B)** Assessment of neutrophil oxidative burst activity using dihydrorhodamine (DHR) flow cytometry assay. Upon stimulation by phorbol 12-myristate 13-acetate (PMA), the patient showed no change in the fluorescence intensity of rhodamine 123 (right panel). This result is consistent with CGD that lacks normal neutrophil oxidative burst activity. The percentage in the histograms indicates the proportion of neutrophils that produced reactive oxygen species (ROS). PMA, phorbol 12-myristate 13-acetate.

ExomeDepth identified a total of 195 CNV calls in the patient, of which 19 were shared with the control and two were localized on sex chromosomes. The CNVs retained for further analysis encompassed 94 deletions and 80 duplications. The proportion of predicted CNVs with a size larger than 1 kilobases (kb) (63.8%) was higher compared to those shorter than 1kb (36.2%). A high Bayes factor of 1,100 was computed for the heterozygous deletion on chromosome 7q11.23 ranging from 73,303,201 to 74,836,526, corresponding to the WBS region (**Figure 2A**). The 1.53Mb deletion involved genes from *NSUN5* to *GTF2IRD2*, including *NCF1*. The second allele of *NCF1* was found to be deleted, resulting in biallelic loss of *NCF1*.

### Impaired Neutrophil Oxidative Burst Activity

In response to PMA, the patient showed profoundly low intensity of rhodamine 123 as compared to a distinct shift in the control (**Figure 2B**). Decreased oxidation of DHR in the patient indicated defective oxidative burst activity of NADPH oxidase.

### Validation of ΔGT in *NCF1*

**Figure 3:**
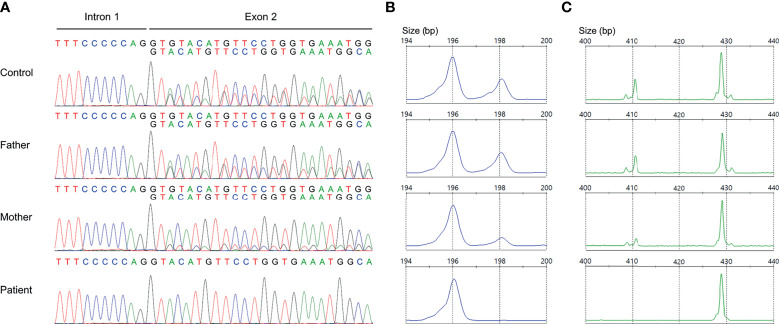
Validation of GT-deletion (ΔGT) in *NCF1*. **(A)** Coamplification of *NCF1* and its pseudogenes at intron 1/exon 2. Overlapping peaks of GTGT-containing *NCF1* and ΔGT-containing pseudogenes were observed in the control and parents. The patient only had a product of *NCF1* pseudogenes, indicating a ΔGT in *NCF1* (NM_000265.7: exon 2: c.75_76delGT: p.Tyr26Hisfs*26). **(B)** ΔGT gene scan. The fragments of *NCF1* and its pseudogenes were depicted by the 198-bp peak and 196-bp peak respectively. A normal ratio of 2:4 was identified in the control and father, reflecting two copies of *NCF1* per four copies of pseudogenes. Meanwhile, the mother had a ratio of 1:5, signifying the carrier of ΔGT in *NCF1*. Only a single peak of *NCF1* pseudogenes was observed in the patient. **(C)** 20-bp gene scan. The 411-bp-peak and 429-bp-peak represented the fragments of *NCF1* and its pseudogenes respectively. Results from 20-bp gene scan corroborated the ratios from ΔGT gene scan. The patient was confirmed to have a biallelic loss of *NCF1*, in which one allele was included in the Williams-Beuren syndrome (WBS) deletion while another allele harbors a ΔGT. bp, base pair.

Nonallele-specific amplification displayed overlapping traces of GTGT-containing *NCF1* and ΔGT-containing pseudogenes in the control and parents (**Figure 3A**). Conversely, only a sequence of *NCF1* pseudogenes was shown in the patient, indicating a ΔGT in *NCF1* (NM_000265.7: c.75_76delGT: p.Tyr26Hisfs*26). ΔGT gene scan detected fragments of two sizes, i.e., 196-bp fragment reflected *NCF1* pseudogenes with ΔGT and 198-bp fragment reflected *NCF1* with GTGT. The proportion of GTGT and ΔGT was found to be 2:4 in the control and father (**Figure 3B**). However, the patient had a single peak of *NCF1* pseudogenes, confirming the absence of wild-type *NCF1*. The mother had a ratio of 1:5, signifying the carrier of ΔGT-containing *NCF1*. In 20-bp gene scan, 411-bp fragment denoted the product of *NCF1* with GTGT and a single 20-bp stretch while 429-bp fragment denoted the product of pseudogenes with ΔGT and a duplication of 20-bp stretch. Ratios of *NCF1* and its pseudogenes obtained from ΔGT gene scan were consistent with that of 20-bp gene scan (**Figure 3C**).

## Discussion

In this study, we reported a case with dual molecular diagnosis of WBS and chronic granulomatous disease (CGD). Repeated infections and formation of skin abscesses in our patient raised the suspicion of an IEI. We performed initial screening for germline short variants associated with IEI using WES, but detected no causative variants matching the clinical presentation. Further analysis involving CNV detection from exome data uncovered a biallelic loss of *NCF1*, suggesting CGD. This finding was corroborated by DHR assay that showed remarkably low neutrophil oxidative burst activity. DHR assay is commonly used to diagnose CGD owing to its quantitative measurement of NADPH oxidase activity and high sensitivity (22). However, abnormal DHR oxidation in complete myeloperoxidase deficiency and Rac2 deficiency may lead to false-positive CGD diagnosis (23, 24). Thus, genetic analysis is useful to confirm the diagnosis of CGD.

Oxidative burst activity of NADPH oxidase, which involves ROS production is essential for the elimination of invading pathogens (25). Defects in one of the five subunits of NADPH oxidase will impair the release of ROS, resulting in CGD (26). Due to ineffective clearance of microbes, CGD patients are predisposed to recurrent infections and excessive inflammation (22). This IEI was estimated to affect 1/200,000 to 1/250,000 newborns, with majority of the cases caused by X-linked *CYBB* mutations (27). Monogenic defects in *CYBA*, *CYBC1*, *NCF1*, *NCF2* and *NCF4* led to the less common form of CGD inherited in autosomal recessive pattern (28, 29).

**Figure 4:**
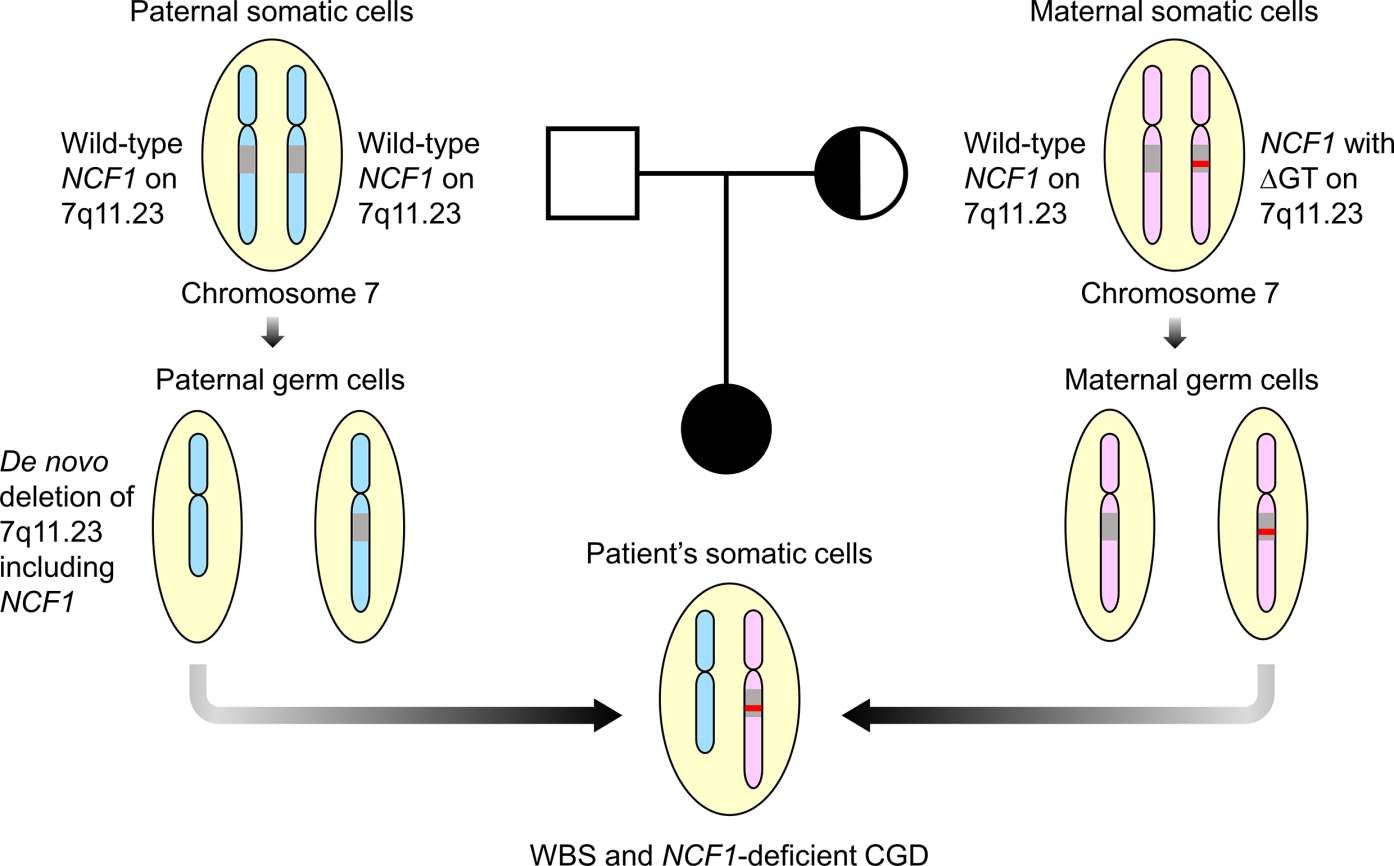
Familial segregation analysis. The patient was postulated to inherit a paternal allele with *de novo* 7q11.23 deletion involving *NCF1* and a maternal allele with ΔGT-containing *NCF1*, resulting in Williams-Beuren syndrome (WBS) and *NCF1*-deficient chronic granulomatous disease (CGD). Both parents were well with no features of WBS and CGD. Square symbol indicates male, half-filled circle indicates female carrier of *NCF1* with ΔGT and filled circle indicates female affected by WBS and CGD. The diagram was not drawn to scale. ΔGT, GT-deletion; WBS, Williams-Beuren syndrome; CGD, chronic granulomatous disease.

Mutation detection in *NCF1* can be tricky due to the presence of two pseudogenes that share at least 98% of DNA sequence similarity with the gene (30). Prominent differences, including GTGT at exon 2 and single 20-bp stretch at intron 2 distinguished *NCF1* from its pseudogenes that contain a ΔGT and duplicated 20-bp stretch respectively (31). Taking these two features into account, Dekker et al. had established a reliable gene scan method to determine the ratio of *NCF1* and its pseudogenes (21). The ΔGT and 20-bp gene scans revealed *NCF1* deficiency caused by a ΔGT in our patient as opposed to a normal ratio of 2:4 and a carrier ratio of 1:5 in the father and mother respectively. Deletion of GT in *NCF1* led to a frameshift followed by premature termination at amino acid residue 51, generating a truncated p47^phox^. Unusually high incidence of ΔGT in unrelated patients was thought to arise from unequal crossing-over between *NCF1* and its highly homologous pseudogenes (31, 32). Clinical symptoms of WBS and CGD in the patient can be explained by a WBS deletion involving one *NCF1* allele and a ΔGT in the second *NCF1* allele. As the mother was found to be the carrier of ∆GT-containing *NCF1*, we hypothesized that the patient inherited the *de novo* 7q11.23 deletion including *NCF1* from her father (**Figure 4**). The genetic diagnosis has enabled our patient to be treated with continuous prophylactic antibiotics and antifungal to prevent severe infections.

Patients with defective NADPH oxidase are increasingly susceptible to infections caused by catalase-positive microorganisms (27). This is because microbes with catalase are able to break down both host and microbial hydrogen peroxide and escape from the oxidative killing. On the contrary, catalase-negative microorganisms seldom caused infections in CGD patients as they were killed by their self-produced hydrogen peroxide. Interestingly, we detected the growth of catalase-negative *Streptococcus* sp. in our patient in addition to catalase-positive bacteria, i.e., *Escherichia coli* and *Salmonella typhi*. The pathogenicity of *Streptococci* in CGD can be explained by some strains that produced no hydrogen peroxide (33, 34). Hence, the possibility of getting infected by catalase-negative microbes should be taken into consideration despite being rare in CGD patients.

Misalignment of segmental duplications on chromosome 7q11.23 mediates nonallelic homologous recombination during meiosis, resulting in WBS deletion (35). Cases with a 1.5Mb deletion are more common than those with a larger deletion of 1.8Mb (36). Diagnosing WBS using fluorescence *in situ* hybridization with an *ELN*-targeting probe hampers the detection of deletion size (37). This limitation was then overcome by chromosomal microarray and multiplex ligation-dependent probe amplification (38, 39). Alternatively, we used read depth data from WES to call CNVs, revealing a heterozygous deletion of 1.53Mb on chromosome 7q11.23. The deletion breakpoints were predicted within *NSUN5* and *GTF2IRD2*, affecting 27 genes, including *ELN* and *NCF1*. Phenotypic consequences were identified in some of the deleted genes. As reported by Curran et al., haploinsufficiency of *ELN* was responsible for the pathogenesis of aortic stenosis (40). This heart abnormality was noted in our patient, whom had single copy of *ELN*. Arterial stiffening, possibly due to elastin insufficiency, may increase the risk for cardiovascular events (41, 42). Contrastingly, hemizygosity for *NCF1* generated lower oxidative stress, serving as a protective factor for hypertension (43). Biallelic pathogenic mutations in *NCF1* will lead to autosomal recessive CGD (20). Stenton et al. recently revealed a causal link between homozygous mutations in *DNAJC30* and autosomal recessive Leber’s hereditary optic neuropathy in humans (44). This feature has yet to be reported in WBS patients. Understanding the potential impacts of genetic variants help to predict the disease progression and tailor a proper therapeutic treatment.

Dual molecular diagnosis caused by two disease loci within a patient was less widely recognized before the adoption of NGS. According to a retrospective analysis of exome by Smith et al., patients with clinically complex phenotypes were more likely to have multiple genetic diagnoses (45). This finding suggests the need for reevaluation of existing NGS data and additional laboratory screening in patients with phenotypic complexity. Our study described the first case from Southeast Asia, whom was diagnosed with WBS and *NCF1*-deficient CGD using WES. Until now, only six patients were reported with similar dual diagnosis (46–50). Despite *NCF1* hemizygosity conferred a lower risk for hypertension, Stasia et al. reported one *NCF1*-deficient patient suffering from high blood pressure (49). Hence, regular medical evaluation is crucial to detect the unexpected medical conditions and initiate a timely regimen.

There are several limitations in this study. Firstly, WES impedes the detection of true positive variants in genes with pseudogenes and highly repetitive sequence due to poor mapping in these regions. Although WES may not be an ideal approach for mutation screening in *NCF1*, it is useful for the identification of exonic and splicing variants in other CGD genes. Additional genetic testing, particularly WGS is required to detect disease-causing deep intronic variants that will be missed by WES. Secondly, uneven coverage resulted from amplification bias in WES may reduce the sensitivity and accuracy of CNV detection. Wide coverage of WGS made it more superior to detect CNVs that affect both coding and noncoding regions. Chromosomal microarray and multiplex ligation-dependent probe amplification can be used to validate CNVs identified from NGS data.

## Conclusion

Our study revealed a case with dual diagnosis of WBS and *NCF1*-deficient CGD. Blended phenotypes of two genetic conditions can get misinterpreted as atypical presentation of a single disease, resulting in misdiagnosis or underdiagnosis. The use of NGS has facilitated the elucidation of multiple molecular diagnoses caused by multilocus genomic variations. Still, NGS has a low variant detection rate in genes with homologous pseudogenes, which can be complemented by Sanger sequencing. This study demonstrated the capacity of WES to detect both germline short variants and CNVs that may provide insights into pathogenesis and optimal treatment options.

## Data Availability Statement

The dataset presented in this study can be found in online repository. The name of the repository and accession number are as follows: https://www.ncbi.nlm.nih.gov/, PRJNA763322.

## Ethics Statement

The studies involving human participants were reviewed and approved by Medical Research and Ethics Committee, Ministry of Health Malaysia. Written informed consent to participate in this study was provided by the participants’ legal guardian/next of kin.

## Author Contributions

AMR and SBM designed the study framework and supervised the study. MYC performed the experiments and data analysis. MYC and AMR drafted the manuscript. RRPR recruited the patient, provided clinical treatments and contributed critical views to the study. All co-authors critically reviewed and approved the final version of the manuscript.

## Funding

This study was funded by a grant from the Ministry of Health Malaysia (NMRR-16-892-31023) awarded to AMR.

## Conflict of Interest

The authors declare that the research was conducted in the absence of any commercial or financial relationships that could be construed as a potential conflict of interest.

## Publisher’s Note

All claims expressed in this article are solely those of the authors and do not necessarily represent those of their affiliated organizations, or those of the publisher, the editors and the reviewers. Any product that may be evaluated in this article, or claim that may be made by its manufacturer, is not guaranteed or endorsed by the publisher.
